# Effect of saline irrigation and plant-based biostimulant application on fiber hemp (*Cannabis sativa* L.) growth and phytocannabinoid composition

**DOI:** 10.3389/fpls.2024.1293184

**Published:** 2024-03-15

**Authors:** Carmen Formisano, Nunzio Fiorentino, Ida Di Mola, Nunzia Iaccarino, Ernesto Gargiulo, Giuseppina Chianese

**Affiliations:** ^1^ Department of Pharmacy, School of Medicine and Surgery, University of Naples Federico II, Naples, Italy; ^2^ Department of Agricultural Sciences, University of Naples Federico II, Portici, Italy

**Keywords:** abiotic elicitors, bioeffectors, phytocannabinoid, secondary salinization, marginal land

## Abstract

Phytocannabinoids represent the hallmark of the secondary metabolism of *Cannabis sativa*. The content of major phytocannabinoids is closely related to genetic variation as well as abiotic elicitors such as temperature, drought, and saline stress. The present study aims to evaluate hemp response to saline irrigation supplied as NaCl solutions with an electrical conductivity (EC) of 2.0, 4.0, and 6.0 dS m^-1^ (S1, S2, and S3, respectively) compared to a tap water control (S0). In addition, the potential beneficial effect of a plant-based biostimulant (a legume protein hydrolysate) in mitigating the detrimental effects of saline irrigation on crop growth and phytocannabinoid composition was investigated. Sodium chloride saline irrigation significantly reduced biomass production only with S2 and S3 treatments, in accordance with an induced nutrient imbalance, as evidenced by the mineral profile of leaves. Multivariate analysis revealed that the phytocannabinoid composition, both in inflorescences and leaves, was affected by the salinity level of the irrigation water. Interestingly, higher salinity levels (S2-S3) resulted in the predominance of cannabidiol (CBD), compared to lower salinity ones (S0-S1). Plant growth and nitrogen uptake were significantly increased by the biostimulant application, with significant mitigation of the detrimental effect of saline irrigations.

## 1 Introduction

Throughout history and even today, the species *Cannabis indica* L. (*Cannabaceae*) known as cannabis is acknowledged for its effectiveness in treating various medical conditions (Grotenhermen and Müller-Vahl, 2012; Alexander, 2016). Its potential in the pharmaceutical field stems from its rich profile of secondary metabolites, such as phytocannabinoids, terpenes, and flavonoids (El Sohly and Gul, 2014; Hanuš et al., 2016; Gorelick and Bernstein, 2017). Berman et al. (2018) identified more than 100 phytocannabinoids, but the focus of biomedical research primarily centers around three main compounds: Δ^9^-tetrahydrocannabinol (Δ^9^-THC), cannabidiol (CBD), and cannabigerol (CBG). The therapeutic applications of these phytocannabinoids and their derivatives are still being explored. It should be pointed out that even the other species of *Cannabis sativa* L., known as hemp, being a multi-purpose crop used by humanity for thousands of years and for various purposes, can be an interesting source of metabolites useful for medical application (Chandra et al., 2017). The biomedical potential of *C. sativa* has been substantially extended beyond the biological profile of Δ^9^-THC. Recent studies have shown the effectiveness of CBD in treating genetic forms of juvenile epilepsy, such as Lennox-Gastaut and Dravet syndromes (Nelson et al., 2020).

Therapeutic metabolites, especially phytocannabinoids, are predominantly found in female inflorescences (Bernstein et al., 2019a). Their production, either directly or in relation to hemp inflorescence development, is influenced by environmental, genetic, and cultivation factors. While Gorelick and Bernstein (2017) touched upon this, comprehensive information is still limited.

Multiple studies have noted a genotypic influence on cannabinoid production (Jankauskiene et al., 2017; Pieracci et al., 2021; Shiponi and Bernstein, 2021; Danziger and Bernstein, 2021a). Additionally, specific agronomic practices, notably plant architecture manipulation (Danziger and Bernstein, 2021b), plant density (Vera et al., 2004, 2010; Tang et al., 2017; Danziger and Bernstein, 2022), fertilization (Tang et al., 2017), and irrigation (García-Tejero et al., 2014; Pejić et al., 2018), significantly affect the cannabinoid profiles of both medical cannabis and industrial hemp.

Concerning the hemp response to fertilization, recent research has explored the impact of primary macro-elements, including nitrogen (N), phosphorus (P), and potassium (K).

A recent study by De Prato et al. (2022) examined the growth and cannabinoid production of tropical/subtropical hemp varieties under tropical daylengths, temperatures, and different nitrogen rates. The concentrations of cannabinoids, specifically THC, CBD, and CBN, were found to increase with longer daylengths, leading to an extended vegetative phase characterized by heightened photosynthetic activity that with the N supply contributed to increased production of primary and secondary metabolites (De Prato et al., 2022). On the other hand, Saloner and Bernstein (2021 observed that increasing nitrogen levels significantly reduced the concentration of several acidic phytocannabinoids (THCA, CBDA, THCVA, CBGA, and CBCA) in inflorescences of medical cannabis grown under controlled environmental conditions. They found similar outcomes when increasing potassium (Saloner and Bernstein, 2022). Moreover, these authors documented a bell-shaped accumulation curve of phytocannabinoids like Δ^9^-THC, CBD, and CBN in inflorescences in response to nitrogen levels, with diminished values at both the low and high ends (Saloner and Bernstein, 2021). Concerning phosphorus, Shiponi and Bernstein (2021) found that its nutritional levels had varied dose-dependent impacts on the cannabinoid profiles of two medical cannabis genotypes.

Environmental factors also play a role in determining the phytocannabinoid profile of *C. sativa*. These include light spectra (Danziger and Bernstein, 2021c); choice of growing media (Caplan et al., 2017); drought stress, which increases concentrations and yields of main phytocannabinoids in drought-affected hemp compared to adequately irrigated plants (Caplan et al., 2019); and salinity stress, which leads to decreased cannabinoid concentrations as NaCl levels rise (Yep et al., 2020).

It is well-documented that in plants exposed to abiotic or biotic stresses, enzymatic pathways are induced, altering the content of bioactive secondary metabolites (Gorelick and Bernstein, 2014). Among these stresses, salinity is a prominent one. Due to its unique characteristics, hemp could be an effective alternative for agricultural use on marginal lands where the cultivation of food crops is significantly hindered by salt. However, there is limited research on *C. sativa*’s adaptability to salt stress, and the available studies primarily focus on industrial hemp (Akram et al., 2021; Cheng et al., 2016; Di Mola et al., 2021).

Soil salinity is categorized into two types: i) the primary salinity that occurs naturally due to several processes (intrusion of sea wedge into the aquifer, volcanic activity, marine aerosol, lithogenic processes; etc.); ii) the secondary salinity, also termed “anthropic salinity”, which is largely attributed to agricultural practices, especially irrigation. FAO (2014) reports that approximately 400 million hectares of agricultural land worldwide are affected by salinity. In particular, land irrigated with poor-quality (saline) water, excessive groundwater pumping, or changes in vegetation and land use, such as deforestation, contribute to this issue (Bharti et al., 2012). More than 20% of irrigated lands are affected by salinity (Machado and Serralheiro, 2017) and, if current salinization trends persist, 50% of arable land could be salt-affected by 2050 (Shannon and Grieve, 1999). Saline soils induce drought stress in plants due to low water potential and can trigger specific ion toxicity, nutritional imbalances, oxidative stress, and hormonal imbalances (Akram et al., 2021). For glycophyte plants, the implications of saline stress include growth reduction, decreased yield, and in extreme conditions, also death. Salinity can also influence product quality, either positively or negatively, depending on various factors such as the intensity and duration of salt stress, typology of salt, phenological phase during stress exposition, and the plant’s genetic composition.

Exposure to salt stress can lead to a reduction in fiber hemp growth and considerable changes in the plant’s morphology, anatomy, and physiology (Akram et al., 2021), with significant modifications in the lumen of xylem vessels (Guerriero et al., 2017). Additionally, changes in soil salinity have been shown to influence the secondary metabolite profile. Bernstein et al. (2010) reported an increase in the levels of essential oils and carotenoids in sweet basil under varying soil salinity conditions.

Despite the overall detrimental effects of salinity on hemp growth and development, the key advantage of utilizing cannabis in saline environments lies in the ability to allocate different parts of the plant for distinct industrial purposes. For instance, stems can be dedicated to fiber production while inflorescences can be harvested for cannabinoid production.

Recent research has revealed various cropping strategies for mitigating the detrimental effects of salinity. Among these strategies, the use of biostimulants emerges as an innovative and eco-friendly tool that enables cultivation under adverse conditions due to abiotic and biotic stressors (Andreotti, 2020; Dell’Aversana et al., 2020; Del Buono, 2021). The 2019/1009 European Regulation defines the “plant biostimulant” as *a product stimulating plant nutrition processes independently of the product’s nutrient content with the sole aim of improving one or more of the following characteristics of the plant or the plant rhizosphere: i) nutrient use efficiency; ii) tolerance to abiotic stress; iii) quality traits; iv) availability of confined nutrients in soil or rhizosphere*. Plant biostimulants can be categorized as microbial plant biostimulants, which consist of a micro-organism or a consortium of micro-organisms and non-microbial plant biostimulants (Regulation, E.U., 2019/1009). Depending on their origin, du Jardin (2015) categorized non-microbial plant biostimulants as i) humic substances; ii) protein hydrolysate (PH) and other N-containing compounds; iii) amino acid-containing products, seaweed extracts, and botanicals; iv) chitosan and other biopolymers; and v) inorganic compounds.

Much literature reports the beneficial effects of biostimulants on food crop growth and yield. In recent years, they have also been gaining attention as sustainable tools in the cultivation practices of both medical cannabis and industrial hemp. However, to date, few studies have deeply investigated the response of the Cannabis genus to biostimulant application. Kosmidis et al. (2023), in an outdoor pot experiment, evaluated the combined effect of biocompost (vermicompost, alone or mixed with spent mushroom substrate and cattle manure compost) and biostimulant (seaweed extracts) on the development of the root system of hemp (*Cannabis sativa* L.), and they found that the significantly higher values of length density, surface area density, and nitrogen content of roots were observed in the two treatments with the addition of biostimulant. Moreover, in a more recent study, Wise et al. (2024) assessed the effect of four biostimulant solutions (kelp; *Aloe vera* extract; fish hydrolysate; the complex composed of these three biostimulants) on root growth and nutrient utilization. They observed that biostimulant complexes enhanced root development and increased phosphorus and potassium uptake.

A recent study by Malík et al. (2022) evaluated the potential of amino acid-based biostimulants added to nutrition solution in two different hydroponic systems (recirculated nutrient solution and drain-to-waste system) to improve the cannabinoid and terpene profiles in medical cannabis. Supplementation of amino acids modified the concentration of THCA and CBNA in the flowers, but the concentration curves of both cannabinoid acids were similar; the authors suggest that the exact relationship between the content of secondary metabolites and the nutritional supplements remains unclear.

The effects of protein hydrolysate, humic/fulvic acid, arbuscular mycorrhizae fungi biostimulants, and their combinations have been studied on the uptake of Cd, Pb, and Zn, aiming to find strategies to intensify biomass production from cultivars grown on metal-contaminated agricultural soil (Ofori-Agyemang et al., 2024). In a recent study, Di Mola et al. (2021) investigated the application of a legume-derived protein hydrolysate (LDPH) on hemp seeds irrigated with water containing increasing salt concentrations (ranging from 2 to 6 dS m^-1^ in addition to tap water). Their findings indicated that LDPH elicited an increase in seed yield (+38.6% compared to untreated plants) with a particularly substantial increase at salt concentrations of 2 and 4 dS m^-1^, where LDPH-treated plants nearly doubled in production compared to untreated plants. Moreover, there was a notable increase in residual biomass (+24.6%). The ameliorating effect of biostimulants on salt stress has also been investigated in other horticultural crops. El-Nakhel et al. (2022) reported that the application of LDPH mitigated the detrimental impacts of high salinity (EC levels up to 6 dS m^-1^) in spinach plants. Lucini and colleagues (2015) observed improvements in lettuce growth when treated with a plant-derived biostimulant under saline conditions. However, the application of two different biostimulants on *Diplotaxis tenuifolia* exposed to varying levels of saline stress resulted in variable effects on antioxidant activity and bioactive compounds, including total phenols, carotenoids, and total ascorbic acid.

Therefore, given the limited research on this subject and the potential impact of salinity stress on phytocannabinoid composition, the present study aimed to evaluate the potential beneficial effect of applying a plant-based biostimulant to alleviate the detrimental effects of NaCl saline water irrigation on the production of biomass and phytocannabinoids for medical use in *C. sativa*.

## 2 Materials and methods

### 2.1 Experimental set-up and crop management

Experimental units consisted of mesocosms (Ø=50 cm pots filled with almost 49 L of soil). These were sown on May 18^th^ with Felina 32 hemp (*C. sativa* L.) genotype aiming for a target plant density of 200 pt m^-2^, which equates to approximately 40 plants per mesocosm. An open field experiment was carried out in the facilities of the University of Naples, Dept. of Agricultural Sciences (Portici, Southern Italy; 70 m a.s.l.) using a medium fertile sandy soil (91.0% sand, 4.5% silt, and 4.5% clay, pH of 6.6, organic matter 2.6%, a total N of 1.1 g kg^-1^, C:N of 13, 127.2 mg kg^-1^ of Olsen P_2_O_5_, and 471.8 mg kg^-1^ of assimilable K_2_O) managed to achieve a bulk density of 1.36 Mg m^-3^ within each mesocosm. Saline solutions, prepared by adding 15.7, 35.2, and 54.8 mmol L^-1^ of NaCl to tap water and labeled as S1, S2, and S3, respectively, were used for crop irrigation. These solutions corresponded to electrical conductivities (EC) of 2.0, 4.0, and 6.0 dS m^-1^ and were compared with a tap water control (S0) having an EC of 0.4 dS m^-1^. All irrigation treatments were applied in combination with two levels of biostimulant application: untreated – NoB; and treated – B with Trainer^®^, a legume protein hydrolysate (Hello Nature, Italy) mainly composed of amino acids and soluble peptides previously characterized by Rouphael et al. (2018). A total of eight treatments (4 levels of water salinity-S x 2 levels of biostimulant application-B) were arranged in triplicate over a randomized complete block design consisting of 24 experimental units (4S x 2B x 3 replicates).

Due to the good soil P and K availability, only N fertilization was performed, adding ammonium nitrate (26% N), according to an ordinary rate of 80 kg N ha^-1^. The foliar application of the legume-derived PH biostimulant was carried out four times, approximately every 12 days starting from June 10^th^, at a dose of 3 mL per liter, based on the manufacturer’s recommendations. Simultaneously, the untreated plants were sprayed with tap water.

The amounts of NaCl required for each saline solution were calculated according to the following Equation 1:


(1) $$\text{NaCl concentration}\left(\frac{\text{g Salt}}{\text{L}\;}\right)=0.64\;\left(\frac{\text{g Salt}}{\text{L x dS }{\text{m}}^{-1}}\;\right)\;\text{x}\;\text{EC}\;\left(\text{dS}\;{\text{m}}^{-1}\right)$$

where NaCl concentration represents the quantity of NaCl to be dissolved in 1 L of irrigation water; EC represents the electrical conductivity of the irrigation water, and the coefficient 0.64 is an empirical value derived by past experiments (El-Nakhel et al., 2022). The electrical conductivity (EC) of the irrigation solutions was calibrated using a conductimeter before each application.

For all treatments, the first three waterings were made with tap water to favor good germination and the emergence of seedlings. Then, from June 17^th^ to July 16^th^, mesocosms were irrigated 10 times with saline water according to the experimental design supplying 35.8, 71.7, and 107.5 g of NaCl per mesocosm, for S1, S2, and S3, respectively, with a cumulative water input of 28 liters. Reference ET was estimated by the Hargreaves formula (Hargreaves and Samani, 1985), while crop ET was calculated using the crop coefficients (Kc) proposed by Cosentino et al. (2013) (0.4 from plant emergence to 6^th^ fully expanded leaf, between 0.4 and 1.1 from 6^th^ fully expanded leaf to complete plot covering). Rainfall events were subtracted in the daily calculations. Soil moisture at field capacity (FC) and at wilting point (WP) was 19.1% v/v and 9.1% v/v, respectively, which was estimated using the pedotransfer function by Saxton and Rawls (2006).

The water was manually distributed, and the amount of each irrigation volume was aimed at restoring the readily available water fraction within each mesocosm according to Doorenbos and Pruitt (1979). The refill point for unrestricted growth was fixed according to Di Bari et al. (2004) and Cosentino et al. (2013), who fixed readily available water for fiber hemp at 66% of plant available water.

### 2.2 Soil and plant sampling and analyses

Bulk soil was carefully mixed and then sampled for chemical and physical characterization before the preparation of the experimental units.

The following physicochemical properties were determined on the soil fine fraction (< 2 mm):

soil texture analysis was done using the Boycous hydrometric method; soil pH and EC were measured on 1:2.5 and 1:5 soil (g) water (mL) suspensions, respectively; organic carbon was determined according to the Walkley & Black method (Walkley and Black, 1934); soil organic matter (SOM) was calculated as 1.726 x OC; total nitrogen was determined according to the Kjeldahl method (Bremner, 1965); carbonate was determined according to the Dietrich–Fruhling calcimeter method (Loeppert and Suarez, 1996); and finally, soil P_2_O_4_ content was measured according to the Olsen method, while the ammonium acetate method was adopted for assimilable K_2_O.

The harvest was made on July 19^th^ at full flowering in order to simulate the double use-destination of hemp: inflorescence for medical and nutraceutical purposes and biomass production. For each experimental unit, plants were cut at soil level and weighed in order to obtain the total fresh weight. Total biomass was split into inflorescences, leaves, and stems and weighed; then, for each treatment and replicate, a sub-sample of each part of the plant was oven-dried until constant weight, both for dry matter determination and the analyses reported in the following paragraphs. Plant height and stem diameter were measured over 10 individuals for each experimental unit.

Dried plant tissues were ground separately using a Wiley Mill to achieve a size finer than 20 µm. The Kjeldahl method (Bremner, 1965) was utilized to evaluate total nitrogen in soil and dried plant tissues, which involves mineralization with 96% sulfuric acid in conjunction with potassium sulfate and a trace amount of copper. Separation and quantification of the macro and micronutrients in the dried plant tissues were conducted using ion chromatography (ICS-3000, Dionex, Sunnyvale, CA, USA). This process was paired with a conductivity detector, employing an IonPac CG12 A guard column and an IonPac analytical column for K^+^, Ca^2+^, Mg^2+^, and Na^+^. Moreover, an IonPac AG11-HC guard column and an IonPac AS11-HC analytical column were used for NO_3_^-^, PO_4_^-^, Cl^-^, and SO_4_^2-^, as detailed by Rouphael et al. (2017b).

### 2.3 Extraction procedure and LC-HRMS sample preparation

Leaves and flowers of fiber hemp plants were collected and dried under shadow natural conditions at 25°C. The dried material was ground in a rotary hammer mill and homogenized obtaining an average particle size below 4 mm. Once obtained, the material was stored in sellable and hermetic plastic bags that were stored under dry and dark conditions prior to use.

Powdered leaves and flowers (100 mg for each) were extracted, separately, with 10 mL of methanol ultra LC-HRMS grade, placed in an ultrasound bath at 37 kHz and 800 W, for 15 min, and centrifuged for 10 min at 6000 g. After filtration (PTFE 0.22 µm), the pooled filtrates were evaporated to dryness under vacuum with a rotary evaporator to obtain the dried extracts (S0 L: 13.2 mg; F: 20 mg – S1 L: 21.6 mg; F: 17.7 mg – S2 L: 20.4 mg; F: 18.9 mg – S3 L: 22.7 mg; F: 23.0 mg). The extracts dissolved in methanol ultra LC-HRMS grade (concentration 2 mg/mL) were analyzed by LC-HRMS/MS analysis.

### 2.4 LC–HRMS analysis

All LC-HRMS and LC-HRMS/MS analyses were performed on an LTQ-Orbitrap mass spectrometer equipped with an ESI interface and Excalibur data system (Thermo Fisher Scientific Spa, Rodano, Italy) coupled to a Thermo Ultimate 3000 HPLC system (Agilent Technology, Cernusco sul Naviglio, Italy). The LC-HRMS was carried out on a Kinetex 2.6 µ POLAR C18 100Å (100x3mm) column (Phenomenex, Torrance, CA, USA), using 0.1% v/v of HCOOH in H_2_O (solvent A) and CH3CN (solvent B) as a mobile phase. The gradient elution was optimized as follows: 50% B for 3 min, 50% to 95% B for 20 min, hold for 2 min, followed by 5 min of initial conditions. The total run time, including column wash and equilibration was 28 minutes, with a flow rate of 0.5 mL/min and injection volume of 5 µL. The MS and MSn spectra, in positive and negative mode, were recorded in Data Dependent Acquisition mode, inducing fragmentation of the most intense five peaks for each scan. Source conditions: spray voltage at 3.5 kV (positive mode) and 2.9 kV (negative mode); capillary voltage: 25 V; source temperature: 320°C; normalized collision energy: 25. The acquisition range was *m/z* 150–1500. Although the spectra were recorded in positive and negative mode, only the data obtained in positive mode were taken into account.

### 2.5 Data elaboration and statistical analyses

Plant dry biomass was calculated by multiplying fresh biomass (leaves, stems, and inflorescences) measured for each experimental unit by dry matter percentage measured oven drying the plant sub-samples. Biomass production was recorded as g D.W. m^-2^, taking as reference the mesocosm area, and then converted in Mg D.W. ha^-1^. The N uptake of aboveground biomass was calculated by multiplying dry aboveground biomass by the N content of tissues and then reported as kg N ha^-1^.

A field control, grown in large experimental plots (1000 m^2^), served as a benchmark to compare the crop yields from mesocosms with those achievable under standard conditions in the experimental area.

The following statistical analyses were performed using MS Excel 2013 and SPSS 21 (SPSS Inc. Chicago, USA). A two-way analysis of variance (ANOVA) was performed for biometric parameters using a general linear model. Means were separated according to the LSD test (p< 0.05). A one-way ANOVA was performed to compare the effect of saline irrigation and biostimulant application on cannabinoid-relative concentration in flowers and leaves. In this case, each combination of NaCl salinity level and biostimulant application was considered as a single treatment, accounting for a total of eight treatments. Kolmogorov-Smirnov and Levene tests were carried out to attest normality of distribution and homogeneity of variance, respectively. Logarithmic transformation was applied to studied variables, when necessary, to ensure normality of distribution.

Principal Component Analysis (PCA) (Bro and Smilde, 2014) and ANOVA-simultaneous component analysis (ASCA) (Smilde et al., 2005) were employed to explore the data relative to the phytocannabinoids quantification performed by LC-MS. Both analyses were computed using the PLS toolbox version 8.9 (Eigenvector Research, Manson, USA) under MATLAB environment, version R2018b (MathWorks Inc., Massachusetts, USA). Prior to PCA, the data matrix, made of 16 rows (samples) and 13 columns (measured phytocannabinoids), was submitted to some pre-processing steps. A normalization step was carried out in order to minimize non-sample-related variations. In particular, the norm1 approach was used. Briefly, the area of each cannabinoid was divided by the sum of the areas of all the measured phytocannabinoids within a sample. Then, data was mean-centered and scaled to unit variance (autoscaling). The latter step employs the standard deviation as a scaling factor, thus giving all the phytocannabinoids the same chance to affect the model independently by their absolute values.

## 3 Results and discussion

### 3.1 Crop growth and nutrient assimilation

The mean effect of water salinity and biostimulant application was significant for most of the monitored parameters, while the interaction between these two main factors was not significant. Freshwater (S0) and low NaCl saline (S1) irrigations were not different in terms of total biomass (12.6 Mg DW ha^-1^ on average), stems (6.6 Mg DW ha^-1^ on average), leaves (3.9 Mg DW ha^-1^ on average), and inflorescences (2.4 Mg DW ha^-1^ on average) (**Table 1**). The highest water NaCl salinity level (S3) halved total biomass production, impacting all plant organs with the same magnitude (49% average decrease for stems, leaves, and inflorescences). The S2 irrigation was associated with a clear decrease pattern in plant growth, yet only total and leaf biomass were significantly less than freshwater control. Both plant height and stem diameter were more sensitive to water NaCl salinity compared to other biometric parameters. A pronounced reduction of both parameters occurred at an EC value of 4 dS m^-1^ (S2), and plant height further decreased, shifting from S2 to S3.

**Table 1:** Mean effects of water salinity and biostimulant application on Hemp (cv. Felina 32) yield components.

Water salinity	Total		Stem		Leaves		Inflor.		Height		Diameter	
	Plant biomass (Mg D.W. ha^-1^)								cm		mm	
S0	13.0( ± 1.0)	a	6.6( ± 0.7)	a	3.9( ± 0.4)	a	2.4( ± 0.4)	a	102.9( ± 3.6)	a	4.3( ± 0.2)	a
S1	12.1( ± 2.4)	ab	6.5( ± 1.4)	a	3.7( ± 0.7)	a	1.9( ± 0.7)	a	98.2( ± 5.9)	a	4.3( ± 0.3)	a
S2	9.0( ± 0.6)	bc	4.8( ± 0.4)	ab	2.7( ± 0.2)	b	1.5( ± 0.2)	ab	84.6( ± 4.5)	b	3.6( ± 0.2)	b
S3	6.8( ± 0.8)	c	3.5( ± 0.4)	b	2.3( ± 0.3)	b	1.0( ± 0.3)	b	69.9( ± 2.6)	c	3.1( ± 0.1)	b
Biostimulant												
NoB	8.5( ± 0.9)	B	4.5( ± 0.5)	B	2.6( ± 0.3)	B	1.4( ± 0.3)	B	84.1( ± 4.2)	B	3.6( ± 0.2)	B
B	11.9( ± 1.2)	A	6.2( ± 0.7)	A	3.7( ± 0.4)	A	2.1( ± 0.4)	A	93.7( ± 5.1)	A	4.1( ± 0.1)	A
**Field control**	13.5( ± 1.2)		8.0( ± 1.0)		1.8( ± 0.3)		3.4( ± 0.8)		147.0( ± 7.2)		4.3( ± 0.3)	
Factors (d.f.)	F-statistic											
***WS*** *(3)*	*7.14*		*6.75*		*5.34*		*4.05*		*14.92*		*14.10*	
***BIO*** *(1)*	*10.14*		*8.65*		*8.31*		*5.91*		*6.27*		*12.07*	
***WS x BIO*** *(3)*	*2.87*		*3.07*		*2.42*		*0.87*		*0.97*		*1.66*	
Factors	p-value											
***WS***	*0.003*		*0.004*		*0.010*		*0.026*		*0.000*		*0.000*	
***BIO***	*0.006*		*0.010*		*0.011*		*0.027*		*0.024*		*0.003*	
***WS x BIO***	*0.069*		*0.058*		*0.104*		*0.479*		*0.431*		*0.214*	

Water salinity treatments: S0 freshwater control; S1, S2, and S3 are saline solutions prepared by adding 15.7, 35.2, and 54.8 mmol L^-1^ of NaCl to tap water, corresponding to electrical conductivities of 2, 4, and 6 dS m^-1^, respectively. Biostimulant treatments: NoB is non-treated control; B is plant biostimulation with legume protein hydrolysate. Field control corresponds to hemp grown in large plots under optimal conditions. Means with the same letter (lowercase for water salinity and uppercase for the application of biostimulants) are not different according to the LSD test (P ≤ 0.05). Standard errors (n=6 for water salinity and n=12 for biostimulant) are reported in brackets. The F-statistic represents the ratio of variance explained by tested factors and their interaction to variance within groups. The p-value indicates the likelihood of the results being due to chance.

The total biomass yields for S0 and S1 treatments were in line with the results of other open field experiments carried out in Italy under standard irrigation management. For example, Struik and colleagues (2000) recorded an average biomass yield of 13.7 Mg DW ha^-1^ for the Felina hemp variety. However, the same authors observed a higher stem yield, which accounted for 60-68% of the total biomass contrasting with S0 results from our mesocosm experiment (45-50% of the total biomass). Such a difference could be attributed to varying environmental factors and differences in the experimental setups, as confirmed also by comparing yield data from our S0 pots with our open field control (see MM section). It is worth noting that Tang and colleagues (2017) reported an open-field Felina growth consistent with our findings (5.5 Mg ha^-1^ stems and 2.6 Mg ha^-1^ for inflorescences).

Despite observing significant changes in biomass accumulation across different plant organs, NaCl salinity did not impact the relative allocation of assimilates with stems, leaves, and inflorescences, representing 52%, 31%, and 17% of total biomass (data not shown). Yet, plants from our mesocosm experiment watered with S0 showed a slightly lower relative abundance of stems and inflorescences compared to the field control, likely due to the constraints imposed by the experimental setup.

The dry matter (DM) content of total biomass (average value of 31%) and inflorescences (average value of 29%) remained unaffected by salinity levels. However, the DM contents of stems and leaves were affected differently by increasing water salinity (**Table 2**). Specifically, saline water significantly led to a reduction in the DM content of stems from 35 to approximately 33%. In contrast, leaves showed the highest DM content with S3, which was comparable to S1, and the lowest with S2.

**Table 2:** Mean effects of water salinity and biostimulant application on dry matter content of hemp (cv. Felina 32).

Water salinity	Total		Stems		Leaves		Inflor.	
	%DM							
S0	32.1( ± 0.3)	a	35.2( ± 0.4)	a	29.2( ± 0.4)	bc	29.5( ± 0.4)	a
S1	31.2( ± 0.3)	a	33.0( ± 0.5)	b	29.7( ± 0.1)	ab	28.7( ± 0.8)	a
S2	30.3( ± 0.4)	a	32.9( ± 0.6)	b	28.0( ± 0.4)	c	27.6( ± 0.6)	a
S3	31.4( ± 0.6)	a	32.8( ± 0.8)	b	30.8( ± 0.6)	a	28.4( ± 0.7)	a
Biostimulant								
NoB	31.2( ± 0.3)	A	33.6( ± 0.6)	A	29.2( ± 0.4)	A	28.0( ± 0.5)	A
B	31.3( ± 0.4)	A	33.3( ± 0.4)	A	29.6( ± 0.4)	A	29.1( ± 0.4)	A
Factors (d.f.)	F-statistic							
***WS*** *(3)*	*2.99*		*3.84*		*6.17*		*1.62*	
***BIO*** *(1)*	*0.17*		*0.34*		*1.04*		*2.62*	
***WS x BIO*** *(3)*	*1.31*		*1.23*		*0.39*		*1.15*	
Factors	p-value							
***WS***	*0.062*		*0.030*		*0.005*		*0.223*	
***BIO***	*0.686*		*0.570*		*0.324*		*0.125*	
***WS x BIO***	*0.306*		*0.333*		*0.760*		*0.359*	

Water salinity treatments: S0 freshwater control; S1, S2, and S3 are saline solutions prepared by adding 15.7, 35.2, and 54.8 mmol L^-1^ of NaCl to tap water, corresponding to electrical conductivities of 2, 4, and 6 dS m^-1^, respectively. Biostimulant treatments: NoB is non-treated control; B is plant biostimulation with legume protein hydrolysate. Means with the same letter (lowercase for water salinity and uppercase for the application of biostimulants) are not different according to the LSD test (P ≤ 0.05). Standard errors (n=6 for water salinity and n=12 for biostimulant) are reported in brackets. The F-statistic represents the ratio of variance explained by tested factors and their interaction to variance within groups. The p-value indicates the likelihood of the results being due to chance.

There is limited knowledge about hemp’s resistance to salinity, though recent studies have begun to illuminate its germination process (Sun et al., 2023) and physiological responses at the seedling stage (Zhang et al., 2023) under saline conditions. Our results do not show any major differences between treatments regarding the plant count per square meter (with an average value of 200 ± 22 pt m^-2^). However, as shown in **Table 1**, S1, S2, and S3 limited crop growth by 7%, 30%, and 48%, respectively.

**Figure 1:**
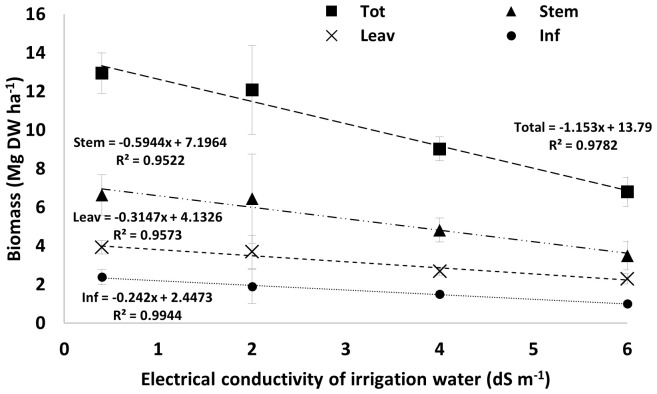
Linear regression models showing the effect of water salinity on total plant biomass and its distribution in stems, leaves, and inflorescences. Bars are standard errors (n=6). EC levels are referred to as water-NaCl solutions with a conductivity of 2, 4, and 6 dS m^-1^.

**Figure 2:**
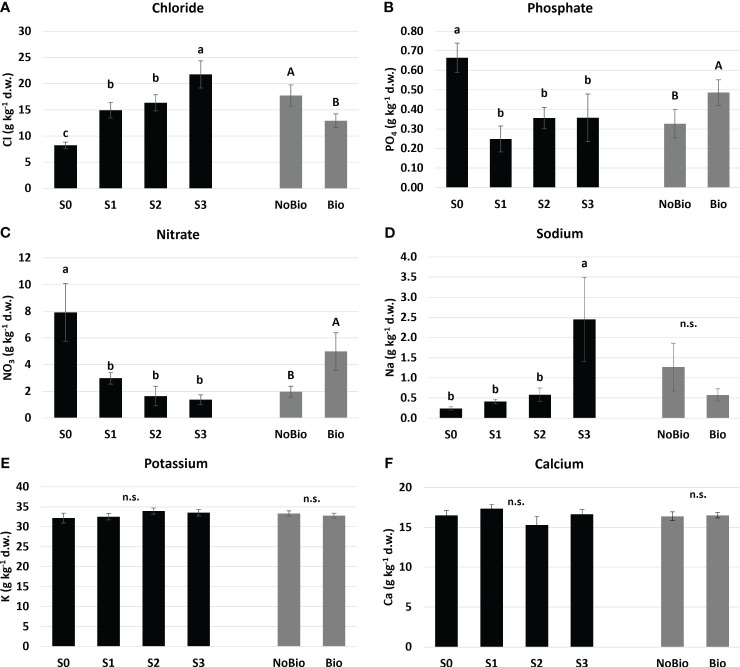
Mean effects of water salinity and biostimulant application on **(A)** Chloride, **(B)** Phosphate, **(C)** Nitrate, **(D)** Soidum, **(E)** Potassium, and **(F)** Calcium assimilation in hemp leaves. Water salinity treatments: S0 freshwater control; S1, S2, and S3 are saline solutions prepared by adding 15.7, 35.2, and 54.8 mmol L^-1^ of NaCl to tap water, corresponding to electrical conductivities of 2, 4, and 6 dS m^-1^, respectively. Biostimulant treatments: NoB is non-treated control; B is plant biostimulation with legume protein hydrolysate. Means with the same letter (lowercase for water salinity and uppercase for the application of biostimulants) are not different according to the LSD test (P ≤ 0.05). “n.s.” indicates non-significant differences. Standard errors (n=6 for water salinity and n=12 for biostimulant) are reported in brackets.

In **Figure 1**, we plotted the average values of total plant biomass and individual organs (stems, leaves, and inflorescences) against levels of water salinity. The regression models fitted to each variable accounted for more than 95% of the data variability and, in all cases, the correlation coefficients were above the critical values for Pearson’s product-moment correlation with 2 degrees of freedom, with p-values ranging from 0.01 to 0.05. Additionally, the slope coefficients of all tested regression functions were highly significant, with p-values between 0.003 and 0.024. This evidence provides a reference point for estimating hemp response to water salinity using the regression function to make some comparisons with other fiber crops. For instance, FAO Paper 29 (Ayers and Westcott, 1989) indicates that flax biomass drops by 25% and 50% occurs with a water EC value of 2.5 and 3.9 dS m^-1^. In contrast, our regression function shows that hemp growth drops by similar percentages at EC values of 3.3 and 6.2 dS m^-1^, respectively. These findings offer initial insights into hemp response to water salinity conditions, suggesting that hemp, being a medium-low tolerance crop, can adapt better to heightened saline levels than flax. It must also be noted that, in addition to osmotic stress, our experiment is subjecting hemp plants to NaCl toxicity, which can cause toxicity and nutrient imbalances (see **Figure 2**), compared to other forms of saline stress. This enhances the value of the information we provided, supporting the idea of identifying hemp as a suitable fiber crop candidate for saline-degraded land.

Biostimulant treatment increased all the productive parameters, raising total biomass, stems, leaves, and inflorescences by 40%, 38%, 42%, and 50%, respectively (**Table 1**). As far as we are aware, the impact of plant-derived protein hydrolysates on the growth dynamics of hemp remains largely uninvestigated. However, these biostimulants proved to be effective in enhancing crop performance also under limiting conditions such as saline and draught stress (Rouphael et al., 2017a), or in low-fertility soils (Di Mola et al., 2020). Our results were coherent with those reported by Di Mola et al. (2021), who showed a 32% boost in total biomass for oilseed hemp (var. Eletta Campana) after the application of protein hydrolysate biostimulants. According to the limited literature on hemp and cannabis cultivation augmented with the addition of plant or animal-derived protein hydrolysates or amino acid mixtures, there are other examples consistent with our research. Wise et al. (2024) demonstrated that fish-derived hydrolysates, either in combination with other compounds or alone, can enhance root growth and nutrient uptake in hemp. Amino acid supplementation under hydroponic conditions (Malík et al., 2022) improved the nitrogen accumulation pattern in the flowers and leaves, also altering the development pattern of cannabis. However, diminished effectiveness of protein hydrolysates on hemp growth was observed under conditions of high metal stress (Ofori-Agyemang et al., 2024).

Crop N nutrition was affected by both water salinity and biostimulant application, though no interaction between these factors was recorded (**Table 3**). Only leaves displayed a variance in N content, with a noticeable dip in N adsorption with S2 and S3 irrigations, bringing down the N content from 2.5% (average value of S1 and S0 treatments) to 2.3% (as average value of S2 and S3 treatments).

**Table 3:** Mean effects of water salinity and biostimulant application on N content and N uptake in hemp tissues (cv. Felina 32).

Water salinity	Total		Stem		Leaves		Inflor.		Total		Stem		Leaves		Inflor.	
	%N								kg N ha^-1^							
S0	1.64(± 0.10)	a	0.50(± 0.05)	a	2.56(± 0.13)	a	3.36(± 0.16)	a	210(± 20)	a	32(± 3)	a	100(± 9)	a	78(± 11)	a
S1	1.50(± 0.05)	a	0.45(± 0.02)	a	2.41(± 0.05)	ab	3.25(± 0.14)	a	180(± 35)	a	29(± 6)	ab	90(± 9)	a	60(± 13)	a
S2	1.44(± 0.09)	a	0.47(± 0.04)	a	2.28(± 0.11)	b	3.03(± 0.07)	a	130(± 12)	b	23(± 3)	bc	61(± 6)	b	47(± 25)	b
S3	1.56(± 0.04)	a	0.58(± 0.03)	a	2.35(± 0.07)	b	3.17(± 0.06)	a	106(± 13)	b	20(± 3)	c	54(± 7)	b	32(± 4)	c
Biostimulant																
NoB	1.42(± 0.04)	B	0.44(± 0.02)	B	2.25(± 0.05)	B	3.11(± 0.09)	A	120(± 13)	B	19(± 2)	B	59(± 6)	B	41(± 6)	B
B	1.65(± 0.05)	A	0.56(± 0.03)	A	2.55(± 0.06)	A	3.29(± 0.07)	A	194(± 18)	A	33(± 3)	A	93(± 9)	A	67(± 8)	A
**Field control**	1.44(± 0.03)		0.50(± 0.04)		2.43(± 0.04)		3.22(± 0.04)		192(± 20)		40(± 5)		43(± 8)		102(± 7)	
Factors (d.f.)	F-statistic															
***WS*** *(3)*	*2.86*		*3.15*		*3.67*		*1.42*		*11.86*		*4.50*		*8.19*		*7.44*	
***BIO*** *(1)*	*18.61*		*14.75*		*23.38*		*2.37*		*29.36*		*28.71*		*18.97*		*13.16*	
***WS x BIO*** *(3)*	*2.06*		*0.82*		*1.85*		*0.57*		*2.45*		*1.36*		*1.97*		*1.10*	
Factors	p-value															
***WS***	*0.070*		*0.054*		*0.035*		*0.275*		*0.000*		*0.018*		*0.002*		*0.002*	
***BIO***	*0.001*		*0.001*		*0.000*		*0.143*		*0.000*		*0.000*		*0.000*		*0.002*	
***WS x BIO***	*0.146*		*0.503*		*0.178*		*0.645*		*0.102*		*0.290*		*0.159*		*0.377*	

Water salinity treatments: S0 freshwater control; S1, S2, and S3 are saline solutions prepared by adding 15.7, 35.2, and 54.8 mmol L^-1^ of NaCl to tap water, corresponding to electrical conductivities of 2, 4, and 6 dS m^-1^, respectively. Biostimulant treatments: NoB is non-treated control; B amendment with legume protein hydrolysate. Field control corresponds to hemp grown in large plots under optimal conditions. Means with the same letter (lowercase for water salinity and uppercase for the application of biostimulants) are not different according to the LSD test (P ≤ 0.05). Standard errors (n=6 for water salinity and n=12 for biostimulant) are reported in brackets. The F-statistic represents the ratio of variance explained by tested factors and their interaction to variance within groups. The p-value indicates the likelihood of the results being due to chance.

Plant N uptake expressed as kg N ha^-1^ mirrors the patterns seen in plant growth. An average total N uptake of 195 kg N ha^-1^ was recorded for S0 and S1 irrigations, with a sharp decrease observed at S2 and S3 (-39% on average). A similar trend was shown by leaves, which contributed to nearly half of the total N allocation, whereas inflorescence and stems showed a more gradual reduction.

Nitrogen uptake values aligned with those recorded by Idilko and colleagues (1997), revealing an N uptake ranging from 142 to 256 kg N ha^-1^ for the cultivar Kompolti. In comparison with our field control, there were no differences in the N content of plant tissues and total N uptake. However, there were notable discrepancies in the N uptake of the different plant organs due to the different biomass partitioning under field conditions.

Biostimulant application significantly increased plant N uptake regardless of water NaCl salinity levels. This was consistently paired with an increase in biomass (**Table 1**) and N concentration (**Table 3**), except for inflorescences. Their N concentration remained consistent between biostimulated plants and the control. These results strongly suggest that the application of protein hydrolysate can enhance nitrogen use efficiency in hemp, even under adverse conditions. The increased assimilatory capacity in biostimulated hemp is likely due to the recognized effect of biostimulants on root development as recently reported by Sifola et al. (2023) in rocket treated with both legume-derived protein hydrolysate and tropical plant extract.

Plant nutrient status was also monitored assessing the mineral profile of hemp leaves. No effect of tested treatments was recorded for several monitored elements, such as for K^+^, Mg^+^, Ca^2+^, and SO_4_^-^ that showed average values in leaves of 33, 3.5, 16, and 3.4 g kg^-1^ (d.w.), respectively, across the experimental units.

As shown in **Figure 2**, Chloride (a) and Sodium (d) were significantly affected by water EC. The first element showed a gradually increasing trend, with S3 displaying the highest Cl^-^ content [almost 20 g kg^-1^ (d.w.)] followed by S1 and S2 (15 g kg^-1^ (d.w.) on average). Conversely, sodium exhibited a significant increase only with S3. These results suggest that the reduction in plant growth under NaCl salinity conditions was not solely due to a decrease in water potential but also due to potential toxicity arising from excessive Chloride assimilation. We can also postulate this process as a possible cause of nutrient imbalance for other anions, specifically Nitrate (**Figure 2C**) and Phosphate (**Figure 2B**). In fact, all EC levels resulted in a lower content of these elements in foliar tissues compared to the S0 control. Our findings are consistent with those reported by Hajiboland (2013) and Niu et al. (2018), which describe how an excess of chloride in irrigation water can disrupt the uptake and translocation of nutrients in hemp plants. Chloride accumulation may competitively inhibit the absorption of essential anions such as nitrates and phosphates, thereby altering the nutritional balance within the plant. High levels of Cl^-^ can also negatively affect plant metabolism by impeding nutrient transporter functions and reducing photosynthetic efficiency.

According to **Figure 2**, the application of biostimulants is linked to a significant decrease in Cl^-^ uptake, which is counterbalanced by an increase in phosphate and nitrate assimilation. This outcome supports the observed trend of enhanced plant growth after biostimulant treatment across various salinity levels and affirms the widely acknowledged effect of biostimulants in ameliorating abiotic stress caused by NaCl salinity. Through mechanisms that involve boosting antioxidant defenses (Calvo et al., 2014), supplying osmoprotective compounds (Sharma et al., 2014), aiding in nutrient uptake, modulating stress hormones, and preserving ion balance, biostimulants enhance plant resilience and counteract nutritional imbalances in saline environments.

### 3.2 LC-MS-based metabolomics analysis of phytocannabinoid content of the investigated *C. sativa* leaves and inflorescences methanol extracts

This section focuses on the qualitative assessment of phytocannabinoids in hemp samples cultivated under varying salt stress conditions. The aim is to explore the potential of different parts of hemp as sources of bioactive compounds for industrial, nutraceutical, and medical applications. The 16 samples submitted to LC-HRMSMS and chemometric analyses were named according to their origin: a group named with the prefix F, which includes eight samples obtained from inflorescences and group L consisting of leaf samples.

An HPLC-HRMS method was developed to be capable of reliably and efficiently identifying a total of 13 compounds with an accuracy error below 10 ppm. HPLC retention times and MS data of compounds in the methanol extracts from leaves and inflorescences of *C. sativa* are listed in **Supplementary Table S1**. As expected, the compound reporting the most intense chromatographic peak (**Supplementary Figure S1**) in all the samples analyzed was CBD, m/z 315.2308 [M + H]+, for which the MS/MS fragmentation pattern was in agreement with the literature data (Chianese et al., 2022), including the fragments corresponding to resorcinol core (m/z 181.12 [M + H]^+^), the p-menthane moiety (m/z 135.15 [M + H]^+^), and partial cleavage of the terpene moiety (m/z 259.10, 235.20 and 193.17 [M + H]^+^) (**Supplementary Table S1**).

The majority of the annotated compounds, identified using standards from prior studies, were identified in the non-polar region, with special emphasis on neutral cannabinoids, including cannabidiol (CBD), cannabidiolic acid (CBDA), delta-9-tetrahydrocannabinol (Δ^9^-THC), cannabinol (CBN), cannabigerol (CBG), cannabielsoin (CBE), cannabielsoic acid (CBEA), cannabichromene (CBC), cannabicyclol (CBL), cannabicitran (CBT), and other phenolic derivatives such as cannflavin A and B and canniprene.

**Figure 3:**
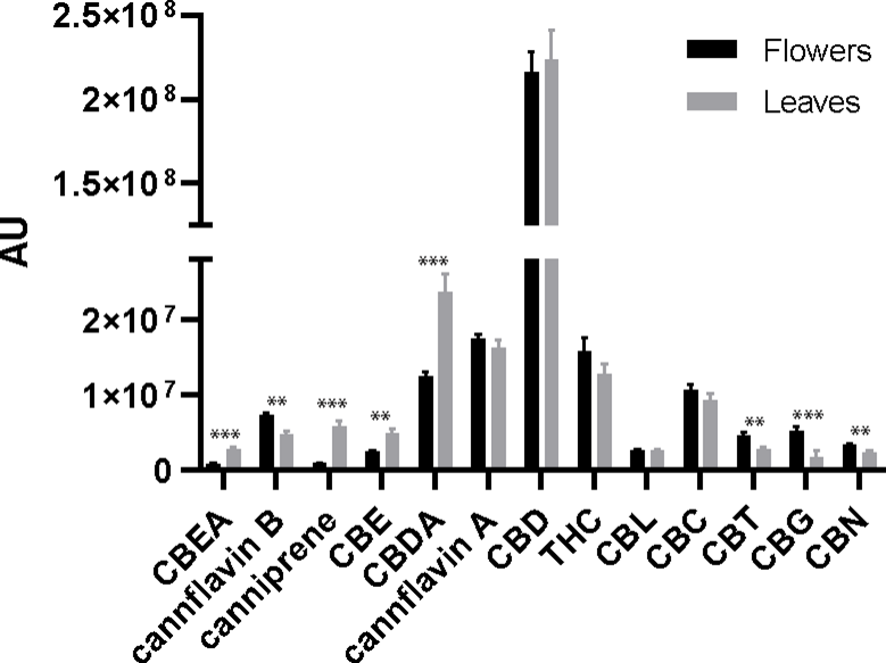
Bar plots and error bars show mean and standard deviations of relative concentrations of cannabinoids calculated from flower and leaf samples (n = 8). The concentrations are expressed in arbitrary units (normalized peak areas). Pair differences are indicated by **P≤ 0.01, and ***P≤ 0.001.

LC-HRMS analysis enabled the identification of 13 phytocannabinoids in the 16 investigated samples. The relative concentrations of the identified cannabinoids in the flower and leaf samples, together with the ANOVA output, are reported in **Figure 3**.

A data matrix, consisting of 16 rows (samples) and 13 columns (measured cannabinoids), was analyzed using an explorative multivariate technique called Principal Component Analysis.

**Figure 4:**
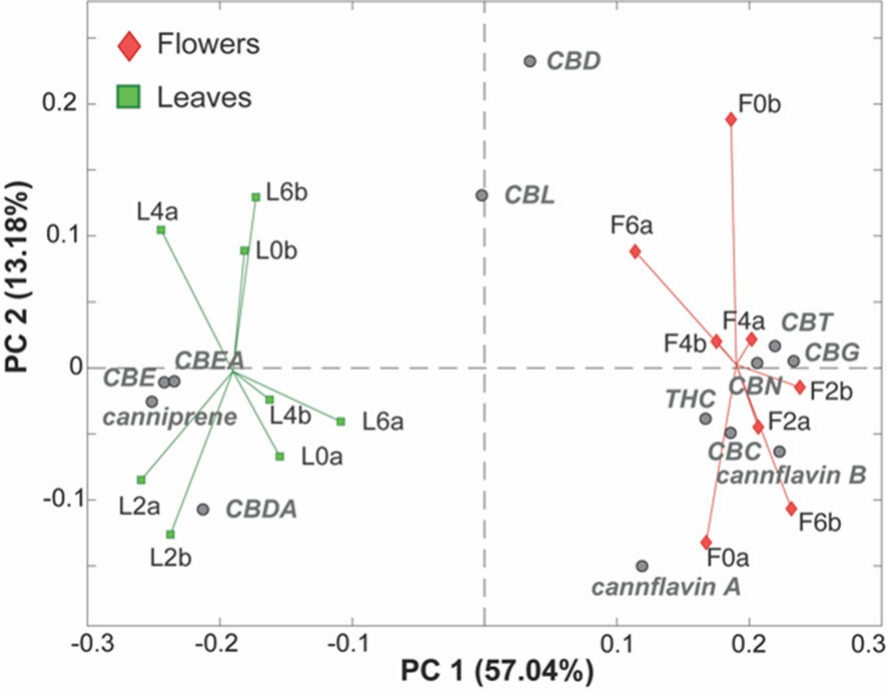
PC1/PC2 biplot of the PCA model of the LC-MS phytocannabinoids data. Samples are colored according to their origin. Leaf samples (green squares), flower samples (red diamonds), measured phytocannabinoids (gray circles).

The resulting PC1/PC2 biplot (**Figure 4**) shows a clear separation between the leaves and inflorescence samples along PC1, accounting for 57.04% of the total variance. This indicates that the origin of the samples is by far the main feature influencing their cannabinoid composition. Specifically, the leaf samples (green squares) exhibit higher levels of CBE, CBEA, canniprene, and CBDA (situated in the left part of the plot), while they presented lower levels of CBT, CBG, CBN, Δ^9^-THC, CBC, cannflavin B, and cannflavin A (located in the right part of the plot), compared to the inflorescence samples.

**Figure 5:**
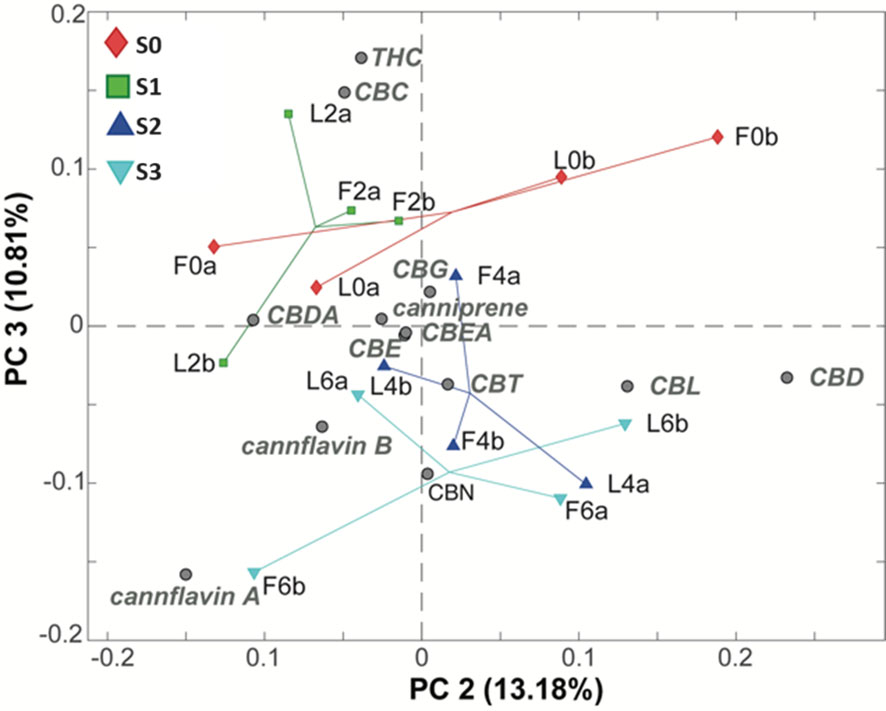
PC2/PC3 biplot of the PCA model of the LC-MS phytocannabinoids data. Samples are colored according to the water salinity. S0 samples (red diamonds), S1 samples (green squares), S2 samples (blue triangles), and S3 samples (cyano triangles).

Upon further examination of subsequent PCs and coloring the samples based on salinity levels, a clear trend regarding this feature was detected in the PC2/PC3 biplot and, especially along PC3, representing 10.81% of the total variance of the dataset (**Figure 5**).

From this plot, it is evident that lower salinity conditions, such as S0 and S1, lead to an increased content of Δ^9^-THC and CBC (located in the upper section of the plot) compared to the group of samples treated with higher water salinity (S2 and S3) which lie, instead, at the bottom of the plot.

Finally, a further inspection of the next PCs facilitated the identification of an interesting trend along PC7 (1.97% of the total variance), as reported in **Figure 5**. In particular, it seems that samples that have been treated with the biostimulant (red diamonds at the bottom of the plot) are characterized by higher levels of CBG and CBDA and lower levels of cannflavin B and CBE, compared to the samples that did not receive the biostimulant.

**Figure 6:**
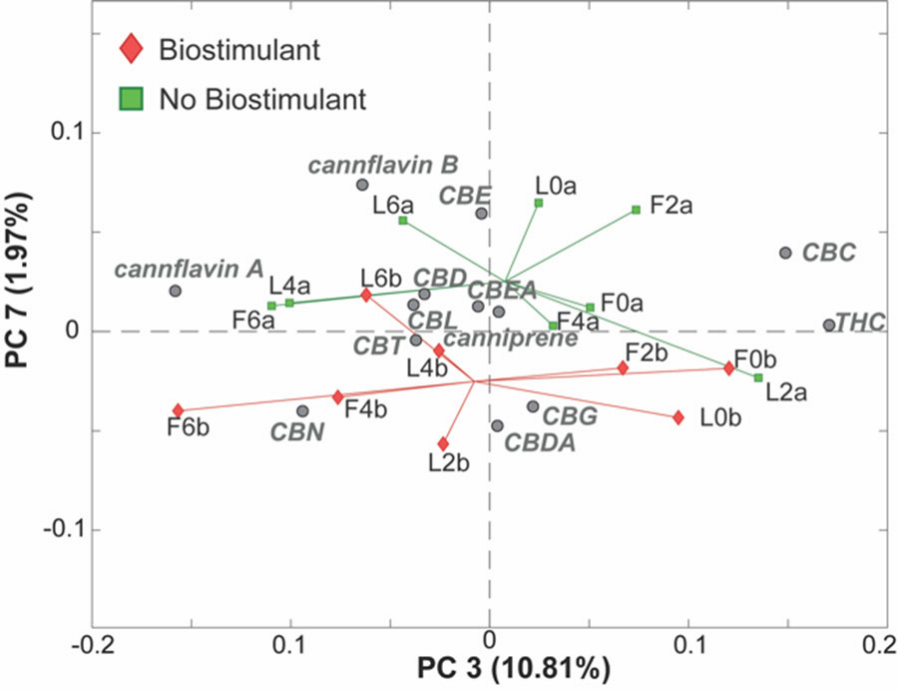
PC3/PC7 biplot of the PCA model of the LC-MS cannabinoids data. Samples are colored according to the presence of the treatment with the biostimulant. Samples treated (red diamonds) or not treated (green squares) with the biostimulant.

Then, in order to partition the variation of the LC-HRMS phytocannabinoids dataset according to the study’s factors (origin, water salinity, and biostimulant), a multivariate extension of the analysis of variance (ANOVA) called ANOVA-simultaneous component analysis (ASCA) (Smilde et al., 2005) was employed. The first factor (Xorigin) corresponds to the different origin of the samples and contains two levels (inflorescence and leaves); the second one (Xsalinity) corresponds to the irrigation water salinity level and it is characterized by 2 levels (low and high salinity). Even though the original classes were S0, S1, S2, and S3, the previously performed PCA model (**Figure 6**) clearly showed only two main clusters corresponding, indeed, to low and high salinity groups. The third factor (Xbiostimulant) is related to the presence or absence of the biostimulant and it also is characterized by 2 levels. The ASCA model also includes three interaction factors (Xorigin x salinity; Xorigin x biostimulant; X salinity x biostimulant) in order to evaluate the presence of potential synergic effects among the three investigated factors.

The ASCA results are reported in **Table 4** and they show, as expected, that the origin of the samples had the biggest effect and explained 54.42% of the total variance, implying that there is a statistically significant variation in the cannabinoid composition of samples obtained from the leaves compared to those obtained from the inflorescences. Furthermore, the salinity effect turned out to be statistically significant even though it explained only 8.62% of the total variance, confirming the results obtained by the PCA. No statistically significant effect was instead found in relation to the presence of the biostimulant. Moreover, no significant interaction effects were found among the investigated factors, meaning they can safely be interpreted independently of each other.

**Table 4:** ASCA-based decomposition of variation according to the three factors included in the experimental design and their two-factor interactions, considering all the investigated samples (n = 16).

Factor	PCs	Variance (%)	*P*-value
*Origin*	1	54.42	*0.0005*
*Irrigation water salinity*	1	8.62	*0.0020*
*Biostimulant*	1	2.50	*0.5325*
*Origin x salinity*	1	2.59	*0.5640*
*Origin x biostimulant*	1	1.78	*0.7840*
*Salinity x biostimulant*	1	2.74	*0.4960*
*Residuals*		27.35	

PCs stands for Principal Components.

The comprehensive understanding of the impact of salt-induced stress on *C. sativa* crops remains incomplete. A recent study investigated salinity sensitivity in an organic potting mix with *C. sativa* in hydroponic and aquaponic solutions during the flowering stage through the addition of up to 40 mM NaCl to a nutrient solution of 1.8 mS cm^-1^. The evaluation focused solely on the Δ^9^-THC/CBD content, revealing a linear decrease in total cannabinoid content with increasing NaCl concentration (Yep et al., 2020). This trend was further validated in 2021 by Anderson et al., who identified optimal fertilizer rates at a low level of 3.5 mM N, observing reduced growth and total cannabinoid concentrations at higher fertilizer concentrations. Their findings highlighted the impact of soil EC and fertilizer rates on CBD concentrations in essential oil hemp, indicating that lower levels of soil EC and fertilizer rates affect CBD more than Δ^9^-THC and CBG (Anderson et al., 2021).

Our study, to the best of our knowledge, presents empirical evidence of how increased salinity levels in *C. sativa* crops can affect not only Δ^9^-THC and CBD but also the content of other minor phytocannabinoids and polyphenols. Notably, our results indicate that CBD concentration is negatively affected by lower soil EC levels (S0 and S1) compared to Δ^9^-THC, while it increases with higher salinity levels (S2-S3). Moreover, the variation in phytocannabinoid concentrations across salinity treatments sheds light on the content of other minor phytocannabinoids with significant bioactivities. Among them, CBC emerges as one of the most abundant phytocannabinoids in cannabis extract, exhibiting a selective affinity for the CB2 receptor and demonstrating modest antinociceptive and anti-inflammatory effects (Pollastro et al., 2018; Udoh et al., 2019). Therefore, our study suggests that implementing effective management practices, combined with phytocannabinoid profiling and testing, could lead to the development of optimized cultivars for industrial or medical purposes.

## 4 Conclusions

Our study has clearly demonstrated the effectiveness of protein hydrolysate biostimulants in mitigating growth limitations imposed by saline water used for irrigating hemp. Furthermore, our findings provide the first insights into biomass hemp response to saline water irrigations with low to moderate EC levels. This information could be useful to exploit marginal areas affected by secondary salinization, where cultivating this versatile crop could yield significant returns compared to other biomass crops. In addition, productive levels recorded for moderate levels of water salinity suggest that this could be a profitable endeavor for farmers.

A condensed economic analysis suggests that hemp cultivation for industrial uses such as bio-composites and biochemicals is economically promising, even under growth-limiting conditions. In Southern Italy, hemp remains profitable, especially in salinized environments where the options for cultivable crops are limited. For comparison, the cultivation of the giant reed as a biomass crop in areas of low fertility yields a gross income of €1,600 per hectare, as reported by Bonfante et al. (2017). The gross income from hemp, based on the biomass yield observed under intermediate salinity conditions in our experiment (almost 10 Mg DW ha^-1^ of biomass) significantly surpasses this figure, given a biomass value of €350 per Mg and variable costs lower than €1000 per hectare. Additionally, processing hemp for biochemicals and phytocannabinoids can further enhance its economic value.

Furthermore, we observed variations in the phytocannabinoid profiles of fiber hemp extracts cultivated under different saline stress conditions. The metabolite compositions of leaves and flowers from cultivars exposed to varying salinity levels displayed differences in relative phytocannabinoid content, underscoring the influence of growing conditions on various biochemical processes, including those responsible for cannabinoid formation. Methanol extracts from cultivars grown with higher salinity levels (S2-S3) predominantly contained CBD), whereas extracts from S0 and S1 treatments exhibited a predominance of Δ^9^-THC. Given the versatile uses of different parts of the cannabis plant for various industrial purposes, it’s evident that salinity can be harnessed to optimize hemp growth for specific phytocannabinoid production from inflorescences.

## Data availability statement

The raw data for LC-MS analysis have been deposited in the Harvard Dataverse and can be found at https://doi.org/10.7910/DVN/JAADNB.

## Author contributions

CF: Investigation, Methodology, Writing – original draft, Writing – review & editing. NF: Conceptualization, Data curation, Investigation, Methodology, Writing – original draft, Writing – review & editing. IDM: Data curation, Formal analysis, Investigation, Writing – review & editing. NI: Data curation, Formal analysis, Writing – original draft. EG: Data curation, Formal analysis, Writing – review & editing. GC: Conceptualization, Funding acquisition, Project administration, Supervision, Writing – original draft, Writing – review & editing.

## Supplementary material

Supplementary Figure 1 LC-HRMS chromatogram of one representative *C. sativa* extract.

Supplementary Table 1 Identified components of the hemp extract were analyzed via LC-HRMS/MS and the main parameters supporting their identification. Compounds are listed in order of LC-HRMS elution. All mass peaks are [M+H]^+^ adducts.
