# Supplementary material for: Effect of saline irrigation and plant-based biostimulant application on fiber hemp (Cannabis sativa L.) growth and phytocannabinoid composition
Source: Front Plant Sci. 2024 Mar 15;15:1293184. doi: 10.3389/fpls.2024.1293184 (PMC10978745; doi:10.3389/fpls.2024.1293184)
Supplement: Supplementary Figure 1 — LC-HRMS chromatogram of one representative C. sativa extract. [file DataSheet_1.docx]

SUPPORTING MATERIAL

Effect of saline irrigation and plant-based biostimulant application on fiber hemp (*Cannabis sativa* L.) growth and phytocannabinoid composition

**Carmen Formisano^1^, Nunzio Fiorentino^2^*, Ida Di Mola^2^, Nunzia Iaccarino^1^, Ernesto Gargiulo^1^, Giuseppina Chianese^1^**

^1^Department of Pharmacy, School of Medicine and Surgery, University of Naples Federico II, Via D. Montesano 49, 80131 Naples, Italy

^2^Department of Agricultural Sciences, University of Naples Federico II, Via Università 100, 80055 Portici, Italy

Figure S1. LC-HRMS chromatogram of one representative *C. sativa* extract


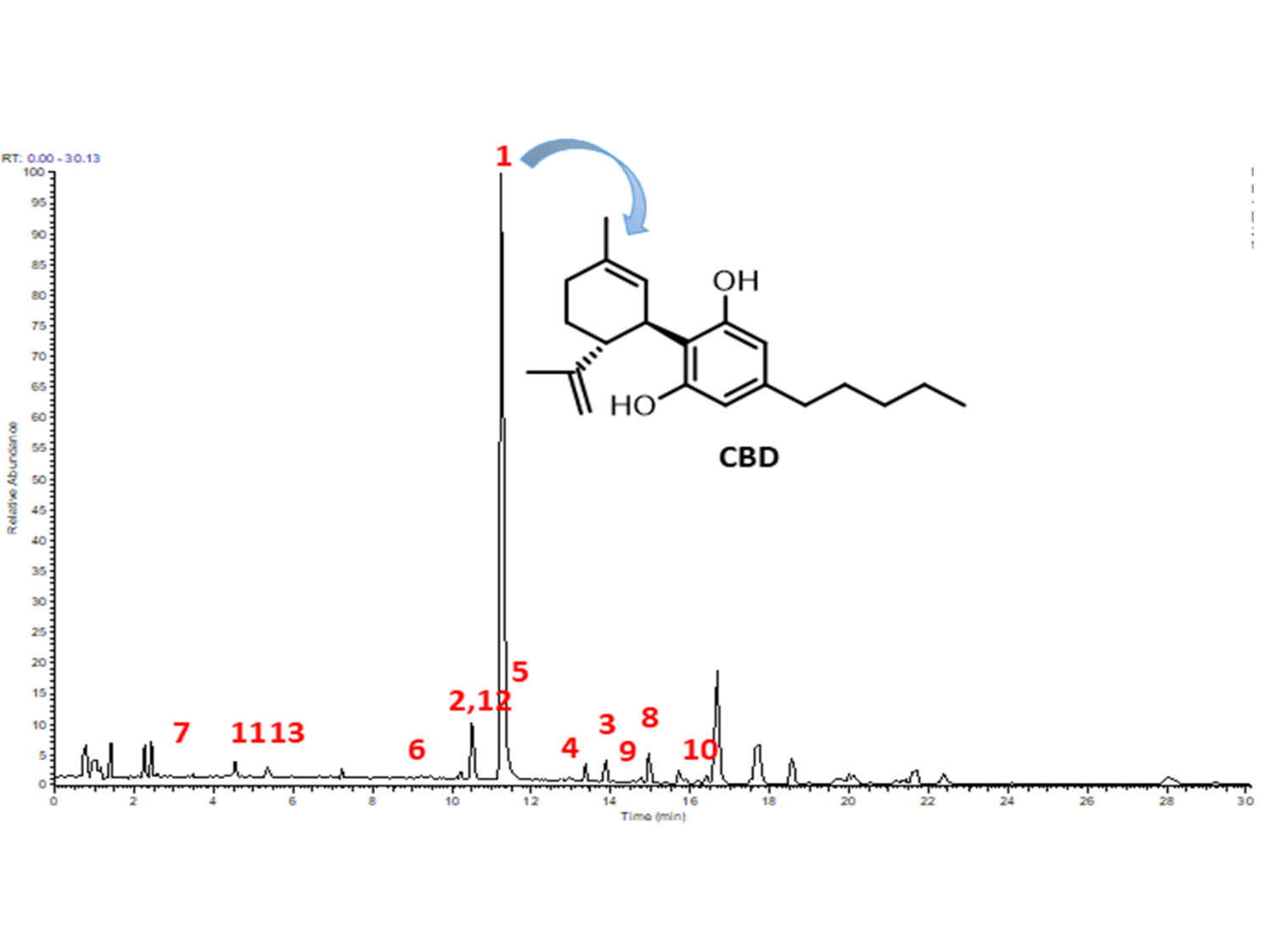


Table S1. Identified components of the hemp extract analyzed via LC-HRMS/MS and the main parameters supporting their identification. Compounds are listed in order of LC-HRMS elution. All mass peaks are [M+H]^+^ adducts.

| Family | Assignment | Formula | Rt (min) | Precursor ion (*m/z*) | Δppm | **Fragments** |
| --- | --- | --- | --- | --- | --- | --- |
| Cannabinoids | Cannabielsoic acid | C_22_H_31_O_5_ | 3.02 | 375.2161 | -0.541 | 357.20, 339.19 |
| Flavonoids | Cannflavin B | C_21_H_21_O_6_ | 4.88 | 369.1319 | -3.806 | 313.07, 217.22, 133.0 |
| Isoprenylated bibenzyl | Canniprene | C_21_H_27_O_4_ | 5.77 | 343.1892 | -3.426 | 329.20, 311.22 |
| Cannabinoids | Cannabielsoin | C_21_H_31_O_3_ | 9.08 | 331.2259 | -2.751 | 313.20, 271.15, 205.14, 181.14 |
| Cannabinoids | Cannabidiolic acid | C_22_H_31_O_4_ | 10.85 | 359.2202 | -5.635 | 341.38, 219.13, 193.22 |
| Flavonoids | Cannflavin A | C_26_H_29_O_6_ | 10.91 | 437.1937 | -6.298 | 381.11, 327.18, 313.16 |
| Cannabinoids | Cannabidiol | C_21_H_31_O_2_ | 11.66 | 315.2308 | -5.187 | 297.24, 259.10, 259.10, 235.20, 193.17, 181.12, 135.15 |
| Cannabinoids | Cannabigerol | C_21_H_33_O_2_ | 11.76 | 317.2462 | -5.785 | 299.23, 261.18, 247.16, 193.12, 181.12 |
| Cannabinoids | Cannabinol | C_21_H_27_O_2_ | 13.33 | 311.1995 | -5.190 | 293.19, 173.59 |
| Cannabinoids | Δ^9^-tetrahydrocannabinol | C_21_H_31_O_2_ | 14.29 | 315.2307 | -5.346 | 259.11, 245.18, 235.19, 193.11, 181.12, 135.16 |
| Cannabinoids | Cannabicyclol | C_21_H_31_O_2_ | 14.96 | 315.2310 | -4.489 | 259.17, 235.17, 193.12 |
| Cannabinoids | Cannabichromene | C_21_H_31_O_2_ | 15.36 | 315.2308 | -5.187 | 259.11, 245.18, 233.21, 193.11, 181.12, 135.16 |
| Cannabinoids | Cannabicitran | C_21_H_31_O_2_ | 16.85 | 315.2310 | -4.299 | 259.17, 193.12 |
